# Serum neurofilament light in familial Alzheimer disease: A marker of early neurodegeneration

**DOI:** 10.1212/WNL.0000000000004667

**Published:** 2017-11-21

**Authors:** Philip S.J. Weston, Teresa Poole, Natalie S. Ryan, Akshay Nair, Yuying Liang, Kirsty Macpherson, Ronald Druyeh, Ian B. Malone, R. Laila Ahsan, Hugh Pemberton, Jana Klimova, Simon Mead, Kaj Blennow, Martin N. Rossor, Jonathan M. Schott, Henrik Zetterberg, Nick C. Fox

**Affiliations:** From the Dementia Research Centre (P.S.J.W., T.P., N.S.R., A.N., Y.L., K.M., I.B.M., R.L.A., H.P., J.K., M.N.R., J.M.S., N.C.F.) and MRC Prion Unit (R.D., S.M.), Department of Neurodegenerative Diseases, UCL Institute of Neurology; Department of Medical Statistics (T.P.), London School of Hygiene & Tropical Medicine, UK; and Clinical Neurochemistry Laboratory, Institute of Neuroscience and Physiology (K.B., H.Z.), the Sahlgrenska Academy at the University of Gothenburg, Mölndal, Sweden.

## Abstract

**Objectives:**

To investigate whether serum neurofilament light (NfL) concentration is increased in familial Alzheimer disease (FAD), both pre and post symptom onset, and whether it is associated with markers of disease stage and severity.

**Methods:**

We recruited 48 individuals from families with *PSEN1* or *APP* mutations to a cross-sectional study: 18 had symptomatic Alzheimer disease (AD) and 30 were asymptomatic but at 50% risk of carrying a mutation. Serum NfL was measured using an ultrasensitive immunoassay on the single molecule array (Simoa) platform. Cognitive testing and MRI were performed; 33 participants had serial MRI, allowing calculation of atrophy rates. Genetic testing established mutation status. A generalized least squares regression model was used to compare serum NfL among symptomatic mutation carriers, presymptomatic carriers, and noncarriers, adjusting for age and sex. Spearman coefficients assessed associations between serum NfL and (1) estimated years to/from symptom onset (EYO), (2) cognitive measures, and (3) MRI measures of atrophy.

**Results:**

Nineteen of the asymptomatic participants were mutation carriers (mean EYO −9.6); 11 were noncarriers. Compared with noncarriers, serum NfL concentration was higher in both symptomatic (*p* < 0.0001) and presymptomatic mutation carriers (*p* = 0.007). Across all mutation carriers, serum NfL correlated with EYO (ρ = 0.81, *p* < 0.0001) and multiple cognitive and imaging measures, including Mini-Mental State Examination (ρ = −0.62, *p* = 0.0001), Clinical Dementia Rating Scale sum of boxes (ρ = 0.79, *p* < 0.0001), baseline brain volume (ρ = −0.62, *p* = 0.0002), and whole-brain atrophy rate (ρ = 0.53, *p* = 0.01).

**Conclusions:**

Serum NfL concentration is increased in FAD prior to symptom onset and correlates with measures of disease stage and severity. Serum NfL may thus be a feasible biomarker of early AD-related neurodegeneration.

There is great interest in testing potential disease-modifying treatments for Alzheimer disease (AD) prior to onset of symptoms. To facilitate this, biomarkers are needed to identify at-risk individuals, stage their disease, and track disease progression.^1^ Ideally, such biomarkers should be noninvasive, inexpensive, and simple to acquire.^2^ Blood-based biomarkers would be very valuable but are more challenging than CSF measures for several reasons, including lower blood concentration of the target analyte, making reliable quantification more difficult.^3^

One promising neurodegeneration biomarker in CSF is neurofilament light (NfL), which increases in a number of neurologic conditions, including AD.^4–8^ NfL can be detected in serum using standard immunoassay formats,^9,10^ but many samples have concentrations below the analytical sensitivity of the methods.^11^ We therefore used a recently developed immunoassay based on the single molecule array (Simoa)^12^ that is 25-fold as sensitive as the previous electrochemiluminescence-based method for NfL.^11^

We measured serum NfL concentrations in familial AD (FAD) mutation carriers and mutation-negative relatives. FAD shares many features, pathophysiologically and clinically, with the more common sporadic form of disease.^13^ FAD mutation carriers have relatively predictable ages at onset,^14^ which allows prospective study of individuals prior to onset of clinical AD. We hypothesized that, with a more sensitive assay, elevated serum NfL would be detectable in FAD mutation carriers prior to symptom onset, and would correlate with disease stage and rate of decline.

## METHODS

### Standard protocol approvals, registrations, and patient consents.

The study was approved by the local research ethics committee and all participants provided written informed consent.

### Participants.

We recruited 48 participants from 24 FAD families to a study at the Dementia Research Centre, University College London, between April 2010 and September 2015. Individuals were eligible if they had either a clinical diagnosis of FAD or a parent with FAD. Eighteen participants were symptomatic, with pathogenic mutations in the *PSEN1* or *APP* genes; 30 individuals were asymptomatic but, by virtue of having an affected parent, were at 50% risk of having inherited a mutation and thereby of developing symptoms at a similar age to their parent (see table e-1 at Neurology.org for family mutations).

For all participants, genetic testing using Sanger sequencing determined the presence or absence of a mutation. Genetic data were provided only to statisticians, ensuring participants and clinicians remained blinded to genetic status; for this reason, it was not possible prospectively to match asymptomatic mutation carriers and noncarriers. Estimated years from symptom onset (EYO) was calculated for mutation carriers by subtracting the age at which the participant's affected parent first developed progressive cognitive symptoms from the participant's current age.

Study procedures included blood sampling, a semi-structured health questionnaire (including exclusion of recent head injury), neurologic examination, cognitive assessment, and volumetric brain MRI, with all assessments completed within 4 months of blood sample collection.

The study was approved by the Queen Square Research Ethics Committee and all participants provided written informed consent.

### Cognitive assessment.

Cognitive assessment included the Wechsler Abbreviated Scale of Intelligence (WASI),^15^ the National Adult Reading Test (NART) (a measure of premorbid IQ),^16^ Recognition Memory Test (RMT) for Faces and Words,^17^ and the Mini-Mental State Examination (MMSE). A close informant was interviewed separately to obtain a collateral history. The Clinical Dementia Rating Scale (CDR)^18^ provided an additional estimate of clinical severity; both global CDR and CDR sum of boxes (SOB) were calculated. Individuals were defined as symptomatic if global CDR was >0 and consistent symptoms of cognitive decline were reported by the participant or an informant.

### Measurement of serum NfL concentrations.

Serum samples were collected, processed, aliquoted, and frozen at −80°C according to standardized procedures. We measured serum NfL using an ultrasensitive immunoassay on the Simoa platform, using the same methodology as described previously.^19^ The lower limits of detection and quantification, as defined by the concentration derived from the signal of blank samples (sample diluent) +3 and 10 SDs, were 0.97 and 2.93 pg/mL, respectively. For a quality control (QC) sample with a concentration of 13.0 pg/mL, repeatability was 14.0% and intermediate precision was 15.7%. For a QC sample with a concentration of 131.8 pg/mL, repeatability was 13.3% and intermediate precision was 13.3%. All measurements were performed by board-certified laboratory technicians in one round of experiments using one batch of reagents.

### MRI acquisition and analysis.

MRI was obtained on 43 of the 48 participants at the time of the blood sample. Five participants were not scanned due to either declining or an inability to tolerate the scan (e.g., claustrophobia). For 33 of the 43 participants with an initial scan, a second scan was performed at a separate visit (mean interval ± SD = 1.3 ± 0.46 years); the other 10 individuals had no second scan due to either leaving the study (n = 5) or it finishing before their second scan date (n = 5).

All scans were performed on the same 3T Siemens (Munich, Germany) TIM Trio scanner using a 32-channel phased array head coil. A sagittal 3D magnetization-prepared rapid gradient echo T1-weighted volumetric MRI (echo time/repetition time/inversion time = 2.9/2,200/900 ms, dimensions 256 × 256 × 208, voxel size 1.1 × 1.1 × 1.1 mm) was acquired. Images were visually checked for artifacts. Four baseline scans and 2 follow-up scans were excluded due to either movement or metallic dental artifact, leaving 39 scans available for baseline volume measurements and 30 pairs of scans for rates of atrophy measurements. Whole brain, ventricular, and hippocampal volumes were calculated using semiautomated methods.^20^ For ventricles and hippocampi, the mean volume from right and left hemispheres was calculated. All volumes were corrected for total intracranial volume (TIV) by dividing a participant's volume by TIV and multiplying by the group mean TIV. Annualized rates of brain, ventricular, and hippocampal volume change during the interscan interval were calculated using the boundary shift interval, a registration-based measure of within-subject volume change.^21^

### Statistical analysis.

The primary objective of the study was to compare serum NfL among symptomatic mutation carriers, presymptomatic mutation carriers, and noncarrier controls. A generalized least squares linear regression model, an extension of the *t* test/analysis of variance model that allows different group-specific residual variances, was used to compare NfL between groups, adjusting for age and sex. Family was included as a random effect to assess any effect of clustering within a family.

Spearman correlation coefficients were calculated to assess the association between NfL and EYO, first across all mutation carriers and then in presymptomatic carriers only and symptomatic carriers only. This rank-based approach, which can be used with bounded variables and is robust to non-normality and outliers, was also used for NfL and cognitive measures, including estimated change in IQ (WASI IQ minus NART-predicted premorbid IQ), recognition memory (an average of scores from RMT faces and RMT words), MMSE, and CDR SOB. We also assessed associations between NfL and the MRI measures. For each association, we first calculated the Spearman coefficient using all available data points, and second using data only from individuals who completed all assessments.

We calculated Spearman correlation coefficients between EYO and each cognitive and imaging measure, including only presymptomatic participants. To allow results to be comparable, these analyses were done using only individuals with all available data points. For all analyses, missing values were assumed to be missing completely at random. Throughout, the threshold for statistical significance was set at *p* < 0.05 (2-tailed) and no adjustment was made for multiple testing.

## RESULTS

**Figure 1:**
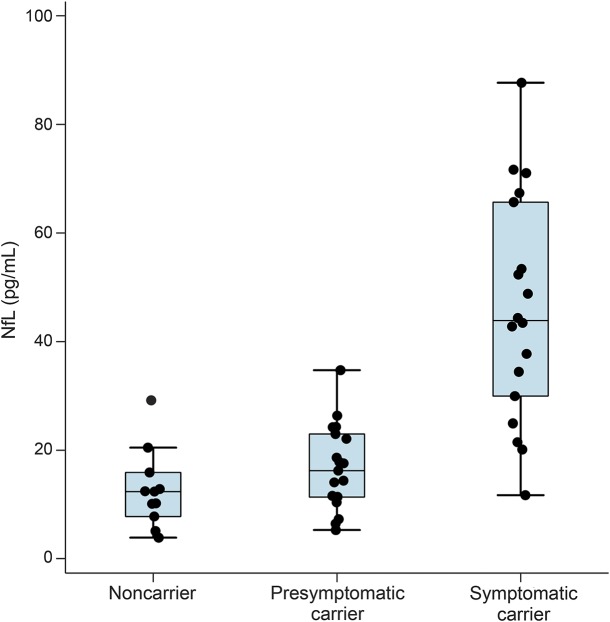
Box and whisker plots for serum neurofilament light (NfL) across the 3 groups The measured unadjusted serum NfL concentrations are shown. Mutation carriers have been divided into those who are symptomatic and those who are presymptomatic.

Participants' demographic details, cognitive scores, neuroimaging measures, and serum NfL values are shown in table 1 and figure 1. Of the asymptomatic participants, 19 were mutation carriers and 11 were noncarriers; noncarriers were used as healthy controls. The mean EYO of the presymptomatic mutation carriers was −9.6 years. Adjusting for age and sex, serum NfL concentration was significantly higher in symptomatic mutation carriers compared with presymptomatic mutation carriers (estimated difference in means 23.2 pg/mL, 95% confidence interval [CI] 13.1–33.2; *p* < 0.0001) and with noncarriers (29.2 pg/mL, 19.3–39.1; *p* < 0.0001). Presymptomatic mutation carriers had significantly higher NfL concentrations than noncarriers (6.1 pg/mL, 1.6–10.5, *p* = 0.007). Allowing for clustering within a family had no effect on results.

**Table 1:**
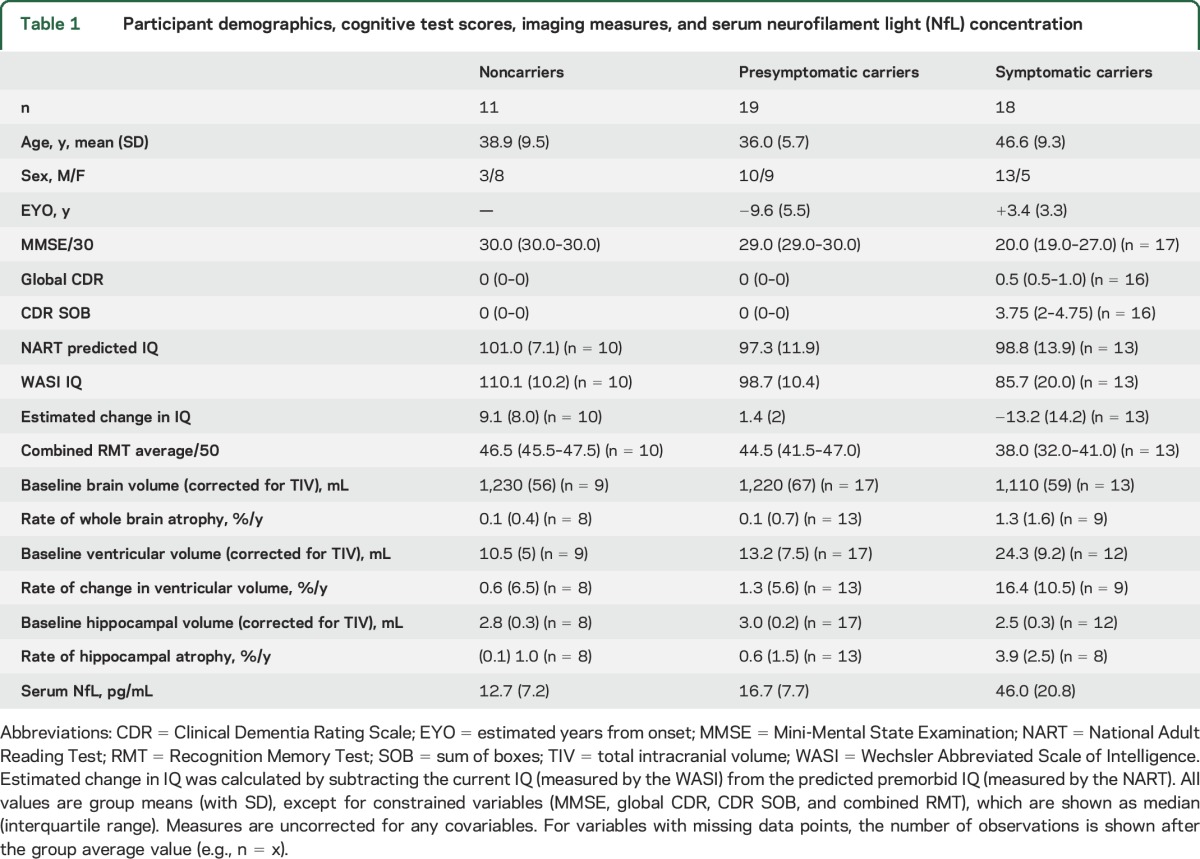
Participant demographics, cognitive test scores, imaging measures, and serum neurofilament light (NfL) concentration

**Figure 2:**
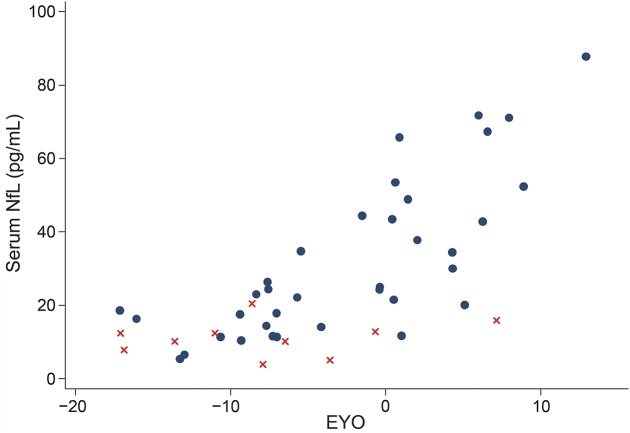
Scatterplot of serum neurofilament light (NfL) against estimated years from symptom onset (EYO) Mutation carriers are represented by dots and noncarriers by crosses. To ensure it is not possible to identify any of the individual asymptomatic participants (based on their EYO) and so determine their mutation status, 2 outlying participants have been removed and a jitter of up to ±2 years has been applied to all remaining participants.

Across all mutation carriers, there was evidence of an association between serum NfL concentrations and EYO (Spearman ρ = 0.81, *p* < 0.0001), with individuals at a later disease stage having higher NfL concentrations (figure 2). Furthermore, this association was significant separately for both the presymptomatic (ρ = 0.55, *p* = 0.01) and symptomatic (ρ = 0.49, *p* = 0.04) groups. A post hoc linear regression analysis in mutation carriers found no statistically significant association between NfL and age, after adjusting for EYO (i.e., disease stage) (*p* = 0.15).

**Figure 3:**
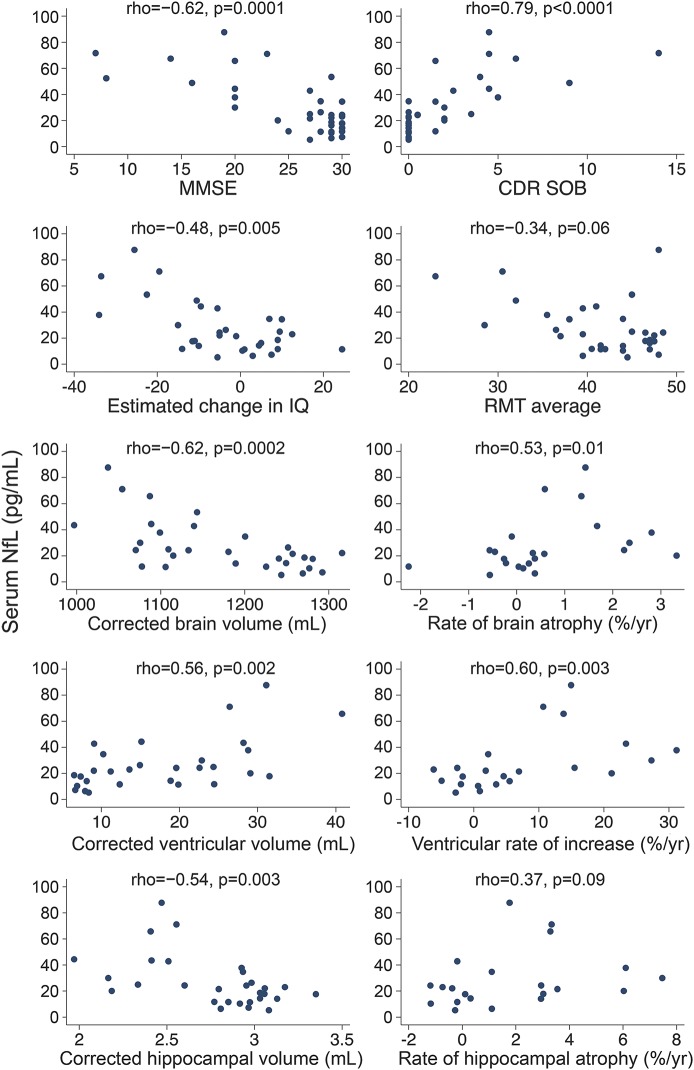
Scatterplots of serum neurofilament light (NfL) against cognitive and imaging measures across all mutation carriers Spearman ρ and the associated *p* value are shown for each scatterplot. Estimated change in IQ was calculated by subtracting the current IQ (measured by the Wechsler Abbreviated Scale of Intelligence) from the predicted premorbid IQ (measured by the National Adult Reading Test). CDR SOB = Clinical Dementia Rating Scale sum of boxes; MMSE = Mini-Mental State Examination; RMT = Recognition Memory Test.

Figure 3 shows scatterplots and Spearman coefficients for serum NfL against cognitive and imaging measures for all mutation carriers, with NfL concentration showing a relatively even distribution throughout the spectrum of disease severity. There were statistically significant correlations between serum NfL and cognitive measures, including MMSE, CDR SOB, and estimated change in IQ, with weaker evidence for a correlation with recognition memory score. In mutation carriers, there was a significant correlation between NfL and cross-sectional neuroimaging measures, including baseline brain volume, baseline ventricular volume, and baseline hippocampal volume. There was also a significant correlation between serum NfL and subsequent rate of change in both brain volume and ventricular volume, but not hippocampal volume. Repeating the analysis including data only from the 19 individuals who completed all assessments did not lead to material change in results, other than for NfL and combined RMT (*p* value changed from 0.06 to 0.6).

For presymptomatic carriers only, there was weak evidence of a correlation between NfL and baseline ventricular volume (ρ = 0.43, *p* = 0.08) and between NfL and CDR SOB (ρ = 0.40, *p* = 0.08), but no evidence of correlations with any other neuroimaging or cognitive measures.

When including only the 13 presymptomatic individuals with serial imaging, there remained a significant correlation between serum NfL and EYO (table 2). However, when assessing the correlations between each of the 6 imaging measures and EYO in the same individuals, none was statistically significant.

**Table 2:**
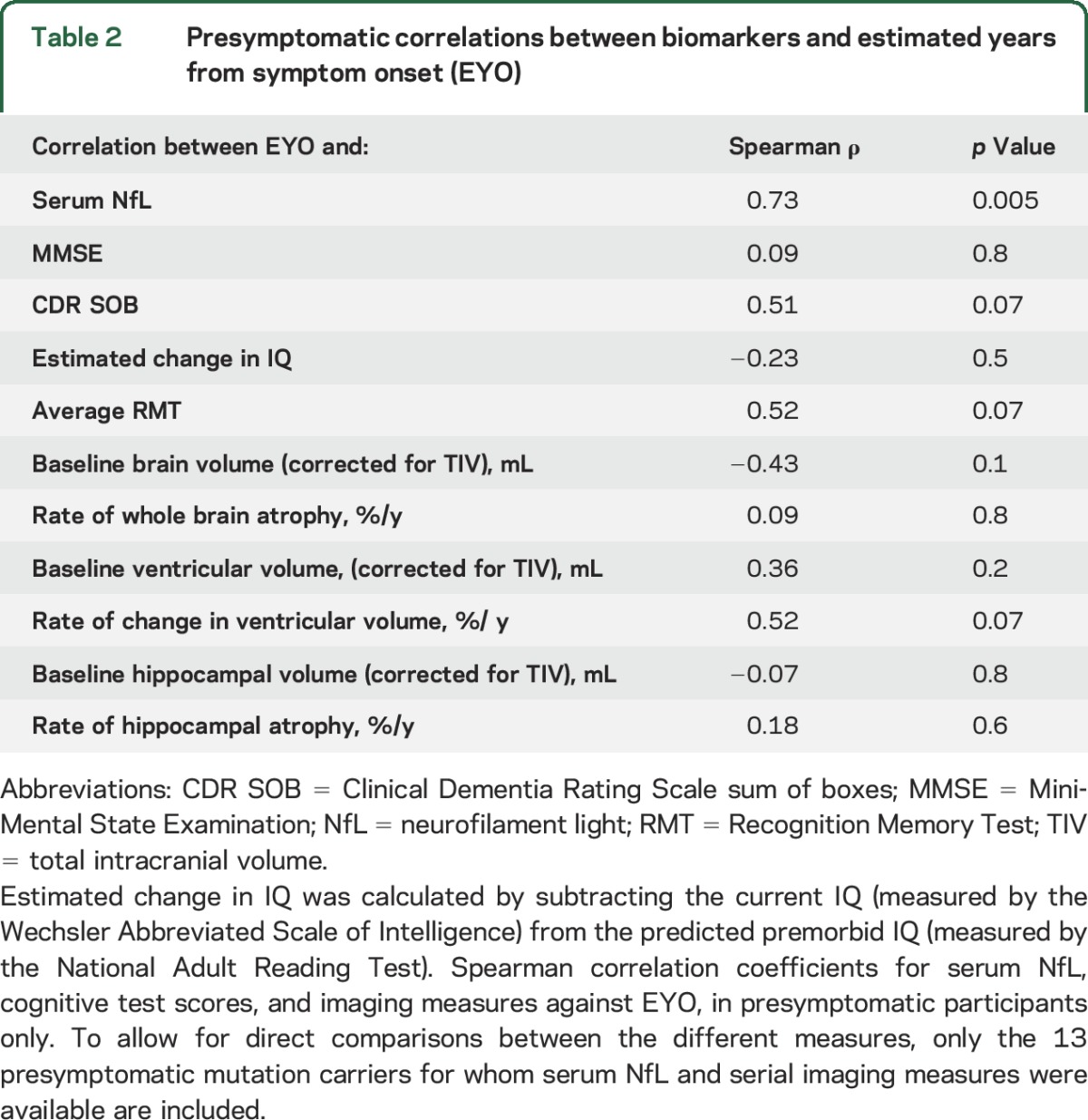
Presymptomatic correlations between biomarkers and estimated years from symptom onset (EYO)

## DISCUSSION

Using an ultrasensitive immunoassay, we found serum NfL concentrations are increased in a group of symptomatic FAD mutation carriers who on average are only mildly clinically affected (median global CDR 0.5); we also found increased NfL concentrations in presymptomatic mutation carriers, who were on average 9 years from their predicted symptom onset. Serum NfL correlated significantly with the estimated years to/from symptom onset (EYO) across all mutation carriers, as well as in the symptomatic and presymptomatic groups separately.

Across all carriers, serum NfL correlated with CDR SOB and several cognitive measures. There was also a correlation between serum NfL and MRI measures of AD-related neurodegeneration, both in terms of cross-sectional volume loss and subsequent rates of atrophy. This suggests serum NfL concentrations may relate to disease severity or rate of progression.

Our serum NfL concentrations for symptomatic FAD are similar to a recent study of sporadic AD that used the same ultrasensitive immunoassay approach.^22^ The mean concentration for our symptomatic group (46.0 pg/mL) (which contained a mixture of mild cognitive impairment [MCI] and AD dementia) lies between their mean values for separate sporadic MCI (42.8 pg/mL) and AD dementia (51.0 pg/mL) groups. However, here we extend previous findings by showing that measurable increases in serum NfL precede the onset of symptomatic disease, and are correlated with predicted time to symptom onset. The observed progressive presymptomatic rise is consistent with proposed models of presymptomatic AD neurodegeneration.^23^ NfL forms an important part of axonal structural integrity, with its rise likely to reflect early axonal breakdown.^24^

Our finding of a presymptomatic increase in serum NfL in FAD mutation carriers contrasts with findings from familial amyotrophic lateral sclerosis (ALS), where no increase was detected until after symptom onset despite symptomatic ALS participants having much higher concentrations than has been detected in either familial or sporadic AD.^25^ This likely reflects differences in the underlying biology and temporal pattern of neurodegeneration in AD vs ALS. ALS is a more aggressive neurodegenerative process in the symptomatic stage, but without the long, gradually progressive presymptomatic phase characteristic of AD. Importantly, atrophy rates are raised in the 5 years before symptoms in FAD and amyloid deposition appears even earlier.^26–28^

The correlation of serum NfL with cognitive measures known to be sensitive to AD-related decline supports the clinical relevance of NfL. While early cognitive changes in FAD most commonly involve episodic memory,^29^ we found that serum NfL correlated more strongly with global cognitive measures than with memory scores. This may relate to the physiologic role of NfL throughout the brain as an essential component of axonal stability, with initial rise possibly reflecting subtle widespread breakdown of neural networks, rather than focal, hippocampal (gray matter) atrophy. The possibility that elevated serum NfL levels more closely reflect global neurodegeneration is also supported by its correlation, across all carriers, with whole brain and ventricular volume loss. This contrasts with findings in other neurodegenerative diseases, including progressive supranuclear palsy (PSP) and frontotemporal dementia (FTD), where disease-specific focal atrophy appeared to be more strongly associated with NfL than whole brain atrophy.^19,30^

It is notable that, while serum NfL correlated significantly with disease stage (i.e., EYO) even when including only presymptomatic participants, imaging and cognitive measures did not. Serum NfL may therefore be a more sensitive marker of early neurodegeneration.

When measured in the CSF of individuals with MCI, NfL has been found to be predictive of subsequent progression to AD dementia,^4^ with a recent meta-analysis showing it to have comparable discriminatory power to the well-established CSF AD biomarkers of Aβ_1-42_, total tau, and phosphorylated tau.^3^ Recent studies comparing NfL measurement in CSF and serum have shown close correlation,^9,10,22^ implying serum NfL may similarly predict subsequent progression, in keeping with our results.

A study in a FAD mouse model, which knocked out the NfL gene, showed that NfL deficiency significantly increased AD-related neurodegeneration, a finding that might suggest a role for NfL in maintaining neuronal structure in patients with AD.^31^ Moreover, in APP/PS1 mice, histopathologic examination found NfL-positive neuritic abnormalities, consistent with increased NfL in AD, signifying underlying axonal damage.^10^ The same study showed serum NfL concentrations increased early in the disease and were closely associated with progression of AD-like pathology. Serum NfL concentrations decreased in response to anti-Aβ immunotherapy, the authors suggesting that serum NfL may serve as a biomarker of treatment response.

There are obvious benefits to identifying AD biomarkers in blood,^2^ with numerous candidates proposed.^32^ However, recent comprehensive meta-analyses of blood-based markers showed only total tau reliably differentiated AD from healthy controls.^3,33^ Moreover, blood tau has only proven useful in identifying AD in established dementia cases, with no evidence that it is useful in earlier disease, and there is often overlap between patient and control groups.^3,32^ Studies attempting to measure blood concentrations of Aβ_1-42_, the other core molecular marker of AD pathology, have so far produced conflicting results, with no strong overall evidence of a difference between AD and controls.^3,32^ Furthermore, even if β-amyloid moieties could be reliably identified and quantified, as cerebral Aβ deposition is thought to plateau some time before symptom onset,^28^ it may not track progression unless very early in disease. By contrast, a marker of downstream neurodegeneration, such as NfL, which may reflect ongoing (global) disease activity, might be useful as a trial outcome measure, from presymptomatic to symptomatic phases. A blood test for neurodegeneration might also be useful clinically in identifying which individuals with cognitive concerns to prioritize for more detailed investigation.

A number of studies investigated plasma or serum profiles in an attempt to identify a pathologic fingerprint of AD, using profiling approaches including proteomics, lipidomics, and transcriptomics.^34–36^ However, poor reproducibility remains an issue when assessing large panels of molecules involved in potentially diverse biological pathways, with several follow-on studies showing negative results.^37–39^ Although our findings find support from previous studies of serum NfL in symptomatic AD,^9,10,22^ it will be important (1) to replicate the presymptomatic findings, before now shown only in mice,^10^ in other at-risk FAD and sporadic AD cohorts (e.g., in amyloid-positive older controls); and (2) to determine the clinical outcomes in these individuals to assess the predictive value and time course of increases in serum NfL.

While our results are encouraging, there are a number of issues regarding the utility of NfL as a biomarker of early AD. While as a group the presymptomatic carriers had higher mean NfL than noncarriers, there was a degree of overlap in observed values. The utility of serum NfL to diagnose presymptomatic AD at the individual level therefore remains uncertain and needs reassessment in independent cohorts. The changes in serum NfL through the course of the disease were analyzed in cross-sectional data only, so it is also not known whether serum NfL tracks progression at an individual level. Also, while our findings support the use of serum NfL as a marker of neurodegeneration in AD, NfL is not a specific marker to AD and has been shown to increase in a number of other conditions, including HIV-associated dementia, PSP, FTD, and ALS.^19,25,30,40^ It may therefore be that serum NfL will be most useful for identifying and tracking AD-related neurodegeneration when combined with a test to confirm underlying AD molecular pathology, e.g., CSF tau/Aβ_1-42_ or amyloid PET.

Our study has limitations. The sample size was not large, owing primarily to the relative rarity of FAD mutations. However, this remains one of the largest single-center FAD cohorts yet reported. For a number of participants, not all cognitive and imaging assessments were completed. However, minimal changes were seen when rerunning the analyses to include only those participants who had completed all assessments. We estimated the age when each mutation carrier would be expected to develop symptoms based on parental age at onset, which is closely associated with actual age at onset^14^; however, this remains a proxy measure, and it is only with longitudinal follow-up that age at onset can be confirmed.

We show, using an ultrasensitive assay, that serum NfL concentration is increased in FAD prior to symptomatic disease, and correlates with the number of years to/from predicted symptom onset. Serum NfL also correlated with neuroimaging and cognitive markers of disease severity. Our findings support the further investigation of serum NfL as an easily accessible biomarker of early AD-related neurodegeneration.

## Supplementary Material

Data Supplement

Accompanying Editorial

## GLOSSARY

AD: Alzheimer disease
ALS: amyotrophic lateral sclerosis
CDR: Clinical Dementia Rating Scale
CI: confidence interval
EYO: estimated years from symptom onset
FAD: familial Alzheimer disease
FTD: frontotemporal dementia
MCI: mild cognitive impairment
MMSE: Mini-Mental State Examination
NART: National Adult Reading Test
NfL: neurofilament light
PSP: progressive supranuclear palsy
QC: quality control
RMT: Recognition Memory Test
Simoa: single molecule array
SOB: sum of boxes
TIV: total intracranial volume
WASI: Wechsler Abbreviated Scale of Intelligence
