# Supplementary material for: Serum neurofilament light in familial Alzheimer disease: A marker of early neurodegeneration
Source: Neurology. 2017 Nov 21;89(21):2167–75. doi: 10.1212/WNL.0000000000004667 (PMC5696646; doi:10.1212/WNL.0000000000004667)
Supplement: Data Supplement [file supp_WNL.0000000000004667_Table_e-1.docx]

Table e-1: Participants’ family mutations

| **Gene** | **Mutation** | **Number of individuals** |
| --- | --- | --- |
| APP | p.Val717Gly | 1 S |
|  | p.Val717Ile | 2 S, 1 AR |
|  | p.Val717Leu | 2 AR |
| PS1 | Intron 4 | 2 AR |
|  | p.Tyr115His | 2 S, 1 AR |
|  | p.Glu120Lys | 2 S |
|  | p.Met139Val | 2 S, 2 AR |
|  | p.Met146Ile | 1 S, 2AR |
|  | p.Leu171Pro | 1 S |
|  | p.Glu184Asp | 1 S, 5 AR |
|  | p.Ile202Phe | 4 AR |
|  | p.His214Tyr | 1 AR |
|  | p.Leu262Phe | 1 AR |
|  | p.Arg269His | 1 S |
|  | p.Arg278Ile | 2 AR |
|  | p.Glu280Gly | 3 S, 7 AR |
|  | ΔE9* | 1 S |
|  | p.Gly394Val | 1 S |

The number of individuals from families with each mutation is given, divided in to either symptomatic (S) or asymptomatic but at risk (AR). Details relating to how many at risk participants for each mutation were mutation carriers is not given to ensure it is not possible for any at risk individual to attempt to deduce their mutation status. * The exon 9 deletion (NM_000021.3:c.869-1G>T; p.Ser290Cys;Thr291_Ser319del) commonly referred to as ΔE9.
