# Supplementary material for: Serum neurofilament light in familial Alzheimer disease: A marker of early neurodegeneration
Source: Neurology. 2017 Nov 21;89(21):2167–75. doi: 10.1212/WNL.0000000000004667 (PMC5696646; doi:10.1212/WNL.0000000000004667)
Supplement: Accompanying Editorial [file supp_89_21_2167_v2_index.html]

Serum neurofilament light in familial Alzheimer disease — Accompanying Editorial 

# Serum neurofilament light in familial Alzheimer disease

## Accompanying Editorial

**Neurology® data supplements are not copyedited before publication. Published editorials and translations have been copyedited.  
 © 2017 American Academy of Neurology.  
  
 Files in this Data Supplement:**

- Accompanying Editorial - PDF
