# Supplementary material for: Serum neurofilament light in familial Alzheimer disease: A marker of early neurodegeneration
Source: Neurology. 2017 Nov 21;89(21):2167–75. doi: 10.1212/WNL.0000000000004667 (PMC5696646; doi:10.1212/WNL.0000000000004667)
Supplement: Accompanying Editorial [file supp_WNL.0000000000004667_WNL.0000000000004675.pdf]

EDITORIAL

Neurofilaments in blood

(Almost) facing clinical application

Michelle M. Mielke, MD, PhD
Markus Otto, MD

Correspondence to Dr. Mielke: Mielke.Michelle@mayo.edu

Neurology® 2017;89:1–2

Neurodegeneration is a critical pathophysiologic process of Alzheimer disease (AD) and related dementias, and correlates better with cognitive symptoms than the sole presence of pathologic proteins such as β-amyloid or α-synuclein. Thus, the identification of a biomarker that tracks with neurodegeneration is critical for following disease progression from the preclinical through the clinical phases and assessing rate of progression or therapeutic utility. The identification of a blood-based biomarker of neurodegeneration would be the Holy Grail. Compared to neuroimaging CSF collection, a blood draw is much less invasive and costly, has minimal side effects, is available in rural areas, and is feasible and acceptable by patients for serial testing to monitor disease progression and therapeutic response.

Neurofilament light chain (NfL) is one such promising potential blood-based biomarker. NfL is a scaffolding protein of the neuronal cytoskeleton that is highly expressed in large-caliber myelinated axons and involved in axonal structural support, growth, and regulation. After axonal injury, regardless of the cause, NfL is leaked to the interstitial fluid, the CSF, and then the blood, where it can now be measured.[1] Unlike plasma β-amyloid or total tau, there is a high correlation between blood and CSF NfL.[2]

In this issue of *Neurology*®, Weston et al.[3] measured serum NfL in asymptomatic and symptomatic familial AD (FAD) mutation carriers and mutation-negative relatives. Serum NfL levels were higher among the symptomatic mutation carriers compared to the asymptomatic carriers, who had higher levels than the noncarriers. Serum NfL was also associated with estimated years to/from symptom onset (EYO). Further, across all mutation carriers, there were significant associations between serum NfL and cognitive measures, ventricular volume, and hippocampal volume. Longitudinally, over a mean interval of 1.3 years, change in serum NfL was associated with change in both brain total and ventricular volume. However, when the analyses were restricted to just the asymptomatic mutation carriers, these associations were no longer significant.

This study is important for multiple reasons. First, the results suggest that serum NfL may be a marker of AD severity or the rate of disease progression. Second, an increase in serum NfL was associated with EYO among the asymptomatic mutation carriers, suggesting that this marker could be an early indicator of disease progression in the preclinical phrase. Third, at least during the symptomatic phase, change in serum NfL correlated with change in multiple measures of brain volume, suggesting that it can track with disease progression. Finally, the results of this study in FAD correspond to recent studies of serum NfL in late-onset sporadic AD that have shown cross-sectional and longitudinal associations with cognitive decline, cortical thickness, and brain volume.[4]

While the findings are promising, a few additional observations warrant discussion and consideration. Serum NfL tracked more strongly with global cognition and global brain volume measures than more AD-specific measures (i.e., episodic memory and hippocampal atrophy). The authors hypothesized that this finding may relate to the physiologic role of NfL throughout the brain, reflecting widespread rather than local breakdown of neural networks. However, in other neurodegenerative diseases—e.g., frontotemporal dementia (FTD), primary progressive aphasia, and progressive supranuclear palsy (PSP)—serum NfL correlates with focal rather than global atrophy.[5,6]

Further, when the current analysis was restricted to the presymptomatic carriers, serum NfL correlated with EYO but not imaging or cognitive measures. The authors hypothesized that this finding may mean that serum NfL is a more sensitive marker of early neurodegeneration. This remains debatable and studies of larger sample sizes with serial serum NfL are warranted. Of note, a recent study using Alzheimer Disease Neuroimaging Initiative data reported that plasma NfL was associated with cognitive and imaging declines of up to 4 years within the MCI and AD groups, but not the cognitively normal group. Similarly, studies of genetic amyotrophic lateral sclerosis (ALS) have reported a sudden and massive

See page 2167

From the Division of Epidemiology, Department of Health Sciences Research, and Department of Neurology (M.M.M.), Mayo Clinic, Rochester, MN; and Department of Neurology (M.O.), University of Ulm, Germany.

Go to Neurology.org for full disclosures. Funding information and disclosures deemed relevant by the authors, if any, are provided at the end of the editorial.

© 2017 American Academy of Neurology

© 2017 American Academy of Neurology. Unauthorized reproduction of this article is prohibited.

increase that is only observed in the symptomatic phase of the disease,[7] and a study of sporadic ALS reported that NfL levels remain high and stable during clinical progression.[8]

It is also worth repeating that while there were group differences in this study (noncarriers, asymptomatic carriers, symptomatic carriers), there was considerable overlap between groups. This finding is in line with several previous studies showing that serum or plasma NfL is not a diagnostic marker of disease stage (e.g., cognitively normal, mild cognitive impairment, AD) or of dementia type (e.g., AD, PSP, FTD). The one diagnostic exception may be for distinguishing Parkinson disease from rarer atypical parkinsonian disorders.[9] However, this lack of diagnostic prowess is not necessarily negative. There are many uses for biomarkers, and one biomarker will not fit all needs. Blood-based measures of NfL appear to be nonspecific markers of neurodegeneration, which could potentially be used to track disease progression and determine therapeutic effect of disease-modifying drugs.

The results by Weston et al.[3] on serum NfL as a blood-based marker of AD severity and disease progression are promising. The next question is how to translate this research for use at the clinical level. In addition to the extensive approval process for a clinically approved laboratory panel, other steps are needed. First, studies suggest that other plasma measures (e.g., total tau[10]) may also be nonspecific markers of neurodegeneration. A comparison of these potential markers is needed to determine which one, or a combination, is most clinically useful. Second, there is a need to understand what serum NfL and other potential markers look like in the population—their range; associations with age, race, sex, and comorbidities; and intraindividual variation. Understanding these aspects will help fast track serum NfL and other potential blood-based markers into useful clinical practice in both rural and urban settings.

STUDY FUNDING

No targeted funding reported.

DISCLOSURE

Dr. Mielke served as a consultant to Eli Lilly and Lysosomal Therapeutics, Inc. She receives research support from the NIH (R01 AG49704, P50 AG44170, U01 AG06786, RF1 AG55151), Department of Defense (W81XWH-15-1), and unrestricted research grants from Biogen, Roche, and Lundbeck. Dr. Otto served as consultant for Biogen and Axon. He receives research funding from the European commission (Fairpark-II), German Federal Ministry of Education and Research (KNDD-FTLDc), JPND (Prefrontals), Thierry Latran foundation, Foundation of the state Baden-Wuerttemberg, Boehringer Ingelheim Ulm University BioCenter (BIU), and German Research Foundation (SFB 1279). Go to Neurology.org for full disclosures.

REFERENCES

1. Kuhle J, Barro C, Andreasson U, et al. Comparison of three analytical platforms for quantification of the neurofilament light chain in blood samples: ELISA, electrochemiluminescence immunoassay and Simoa. Clin Chem Lab Med 2016;54:1655–1661.
2. Gisslen M, Price RW, Andreasson U, et al. Plasma concentration of the neurofilament light protein (NfL) is a biomarker of CNS injury in HIV infection: a cross-sectional study. EBioMedicine 2016;3:135–140.
3. Weston PSJ, Poole T, Ryan NS, et al. Serum neurofilament light in familial Alzheimer disease: a marker of early neurodegeneration. Neurology 2017;89:2167–2175.
4. Mattsson N, Andreasson U, Zetterberg H, Blennow K. Association of plasma neurofilament light with neurodegeneration in patients with Alzheimer disease. JAMA Neurol 2017;74:557–566.
5. Rohrer JD, Woollacott IO, Dick KM, et al. Serum neurofilament light chain protein is a measure of disease intensity in frontotemporal dementia. Neurology 2016;87: 1329–1336.
6. Steinacker P, Semler E, Anderl-Straub S, et al. Neurofilament as a blood marker for diagnosis and monitoring of primary progressive aphasias. Neurology 2017;88: 961–969.
7. Weydt P, Oeckl P, Huss A, et al. Neurofilament levels as biomarkers in asymptomatic and symptomatic familial amyotrophic lateral sclerosis. Ann Neurol 2016;79:152–158.
8. Lu CH, Macdonald-Wallis C, Gray E, et al. Neurofilament light chain: a prognostic biomarker in amyotrophic lateral sclerosis. Neurology 2015;84:2247–2257.
9. Hansson O, Janelidze S, Hall S, et al. Blood-based NfL: a biomarker for differential diagnosis of parkinsonian disorder. Neurology 2017;88:930–937.
10. Mielke MM, Hagen CE, Wennberg AMV, et al. Association of plasma total tau level with cognitive decline and risk of mild cognitive impairment or dementia in the Mayo Clinic Study on Aging. JAMA Neurol 2017;74:1073–1080.

© 2017 American Academy of Neurology. Unauthorized reproduction of this article is prohibited.
